# Senescence-Induced Chemoresistance in Triple Negative Breast Cancer and Evolution-Based Treatment Strategies

**DOI:** 10.3389/fonc.2021.674354

**Published:** 2021-06-24

**Authors:** Anindita Chakrabarty, Shayantani Chakraborty, Ranjini Bhattacharya, Goutam Chowdhury

**Affiliations:** ^1^ Department of Life Sciences, Shiv Nadar University, Greater Noida, India; ^2^ Independent Researcher, Greater Noida, India

**Keywords:** triple negative breast cancer, chemotherapy, senescence, therapy-induced senescence, senescence-associated stemness, evolution, adaptive therapy

## Abstract

Triple negative breast cancer (TNBC) is classically treated with combination chemotherapies. Although, initially responsive to chemotherapies, TNBC patients frequently develop drug-resistant, metastatic disease. Chemotherapy resistance can develop through many mechanisms, including induction of a transient growth-arrested state, known as the therapy-induced senescence (TIS). In this paper, we will focus on chemoresistance in TNBC due to TIS. One of the key characteristics of senescent cells is a complex secretory phenotype, known as the senescence-associated secretory proteome (SASP), which by prompting immune-mediated clearance of senescent cells maintains tissue homeostasis and suppresses tumorigenesis. However, in cancer, particularly with TIS, senescent cells themselves as well as SASP promote cellular reprograming into a stem-like state responsible for the emergence of drug-resistant, aggressive clones. In addition to chemotherapies, outcomes of recently approved immune and DNA damage-response (DDR)-directed therapies are also affected by TIS, implying that this a common strategy used by cancer cells for evading treatment. Although there has been an explosion of scientific research for manipulating TIS for prevention of drug resistance, much of it is still at the pre-clinical stage. From an evolutionary perspective, cancer is driven by natural selection, wherein the fittest tumor cells survive and proliferate while the tumor microenvironment influences tumor cell fitness. As TIS seems to be preferred for increasing the fitness of drug-challenged cancer cells, we will propose a few tactics to control it by using the principles of evolutionary biology. We hope that with appropriate therapeutic intervention, this detrimental cellular fate could be diverted in favor of TNBC patients.

## Introduction

Senescence, a cellular fate originally discovered in the context of growth arrest of cultured cells, is now being recognized as an essential mediator of many physiological and pathological processes (1, 2). Such contradictory outcomes of senescence are explained on the basis of its dynamic and context-dependent pleotropic effects (3). The cellular plasticity and stemness reprogramming functions of senescence (in co-operation with the microenvironment) are believed to be critical for the emergence of the drug-resistant clones in many cancer types, including breast cancer (BC) (4–7).

TNBC, being one of the more heterogeneous and aggressive subtypes of BC, is frequently treated with conventional chemotherapies (8–16). Although better chemosensitivity compared to the other BC subtypes is a key characteristic of primary TNBCs, patients with residual disease frequently experience tumor relapse (17, 18). Among many factors responsible for TNBC chemoresistance, contributions of cancer stem cells (CSC) and therapy-induced senescence (TIS) are well-accepted (10, 17, 19). According to current evidences these two phenomena are causally associated (4, 5, 20).

Immune checkpoint inhibitor (ICI) and DNA damage-response (DDR)-directed regimens are two fairly recent FDA-approved treatment options available for TNBC (21, 22), benefitting only a very small number of patients (21, 23). Interestingly, similar to chemotherapies, efficacies of both strategies are impacted by TIS (23–26). TIS may well be a universal fate assumed by cancer cells when challenged with different types of drugs. Hence, blocking TIS might be a promising approach for better clinical management of several cancer types, especially that of TNBC (23, 27–37).

In the eyes of evolutionary biologists, cancer is an “open complex adaptive system” with non-linear dynamics, prone to suffer unexpected consequences of any kind of perturbations (38). Cytotoxic chemotherapies, meant to cause the highest amount of cancer cell death, is an “evolutionary unsound” approach. By eliminating the entire sensitive population, chemotherapies release the selective pressure on the unwanted resistant clones, a common evolutionary phenomenon termed as the “competitive release” (38–40). To slow the proliferation of the resistant population, it is necessary to alter its fitness or that of the competing populations (38). In the last part of this review, we claim that TIS is an evolutionary fitter strategy for cancer cells following chemotherapy and will attempt to establish how adaptive therapeutic strategies would help alter the fitness of the senescent cells leading to better therapeutic outcome.

## Senescence and Its Hallmarks

**Figure 1:**
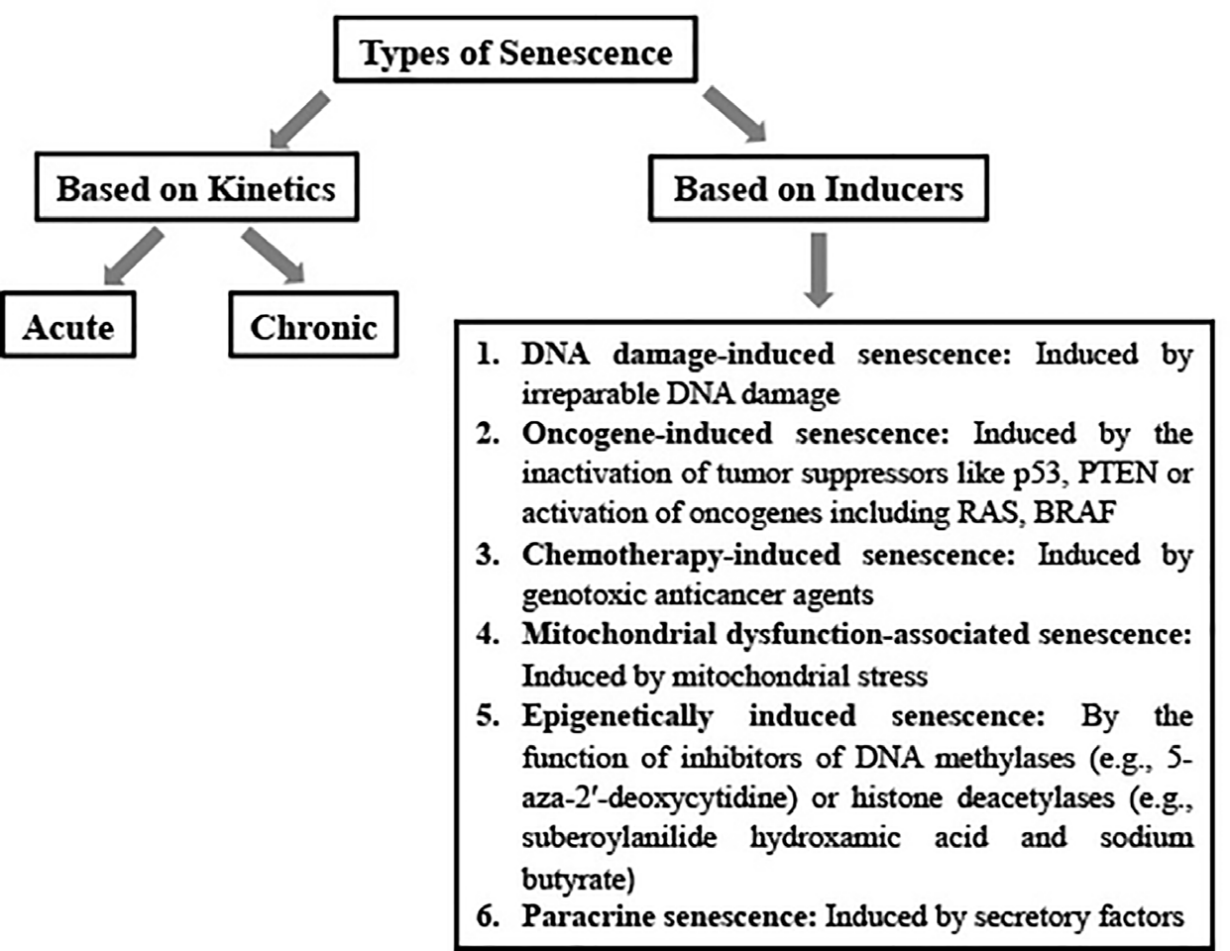
Schematic representation of types of senescence.

Cellular senescence, induced by excessive stress, is a form of cell cycle arrest [irreversible or reversible depending on the context (41, 42); and the references therein]. Senescence is important for numerous physiological and pathological processes such as embryo development, wound healing, tissue repair, atherosclerosis, type 2 diabetes, aging, age-related pathologies, and reduction in regenerative potential following injury (3, 5, 43). It can be acute (programmed, transient) or chronic (non-programmed, sustained) in nature, with the former affecting specific cell population and the latter being non-specific (43, 44) (**Figure 1**). Acute senescence is important for development, wound healing, and tissue repair, chronic senescence, on the other hand, often functions in limiting the proliferation of abnormal cells (43). Senescent cells are highly dynamic and heterogeneous, characterized by not one, but several interesting hallmarks as discussed below (45).

(A) Senescence cells can appear large, flattened, and irregularly shaped, which is attributed to an increased mTOR signaling (46–49) or ATF6a-mediated unfolded-protein response (50–55). In some instances, the plasma membrane protein caveolin-1 is implicated in the morphology and adherence property of senescent cells through the p38 MAP kinase pathway (56, 57).

(B) One of the most appreciated characteristic of senescence is an enhanced activity of the lysosomal senescence-associated beta-galactosidase (SA-βgal) enzyme, due to either increased expression of the gene *GLB1* or increased lysosomal biogenesis (58). SA-βgal cleaves the β-D-galactose residues in β-D-galactosides, such as 5-bromo-4-chloro-3-indoyl-β-d-galactopyranoside (X-gal). In normal cells, SA-βgal is active at pH 4, but in senescent cells, its catalytic activity is detectable at suboptimal pH 6 (5, 43, 59, 60). However, because of the robust signal detected with certain non-senescent, healthy cells in developing embryo, strong SA-βgal-positivity may not necessarily be the best indication of senescence (58).

(C) Accumulation of old and dysfunctional mitochondria due to a reduction in mitophagy is another feature of senescent cells. This is associated with enhanced ROS production through release of mitochondrial enzymes, such as endonuclease G (60–62).

(D) Senescent cells possess decondensed heterochromatin and cytoplasmic chromatin fragments (CCFs), due to a reduction in nuclear structural protein Lamin B1. They attempt to compensate these by forming the senescence-associated heterochromatin foci (SAHF) (63–66).

(E) Upregulation of cell cycle inhibitors p16INK4a, p15INK4b, p21CIP contribute to the senescence-associated growth-arrested phenotype. Furthermore, a chronic DNA damage-response (DDR) pathway activation is detectable in most senescent cells (44). Additionally, senescent cells are apoptosis-resistant due to the upregulation of the BCL-2 family of pro-survival factors (67).

(F) The multifunctional senescence-associated secretory phenotype/SASP is an unequivocal marker of senescence (68). SASP comprises of growth factors, matrix modifying enzymes, cytokines, chemokines, etc. (68–70). The secretory phenotype also includes extracellular vesicles (sEVs) similar to exosomes that participate in cell-to-cell communication through various types of cargos (proteins, lipids, and nucleic acids). Recent reports imply that sEVs are important for tumorigenesis and age-related pathologies (59, 71).

(G) Senescent cells attract, activate, and anchor to immune cells through several cytokines and chemokines (example, IL-6, IL-8, IL-1β, TGF-β, GM-CSF, MCP-1), which ultimately lead to their clearance [(72) and the references therein]. This is called senescent surveillance. Cells of both innate and adoptive immune systems (macrophage, B and T cells, NK cells, mast cells, neutrophils, etc.) are involved in this process [(72) and the references therein]. Molecular features of cell dysfunction and death (DAMPs) produced by senescent cells also facilitate their immune cell-mediated clearance (73). During aging and age-related diseases, senescent cells accumulate in several tissues/organs. Although, this coincides with age-associated impaired or overwhelmed immune system, it is not entirely clear whether dysfunctional immune cells lead to accumulation of senescent cells or senescent cells accumulation leads to immune system failure (72, 74–79). The complex feedback interaction between SASP components and immune cells endows an overall pro-inflammatory and pro-senescent environment in aged animals [(72) and the references therein]. Improving immune-clearance of senescent cells could alleviate many adverse symptoms of old age and other diseases.

(H) Senescence, in some instances, involves autophagy, although the relationship between autophagy and senescence is far from straightforward. Autophagy is a catabolic process important for maintaining cellular homeostasis under conditions of nutrient deprivation. Similar to senescence, autophagy is stimulated by radiation, chemotherapy, telomere shortening, and oncogene activation, shares some common features, and serves similar cytoprotective roles (80, 81). Increased autophagic vacuole formation coincides with heightened SA-β-gal activity in aging fibroblasts (82). Autophagy marker expression overlaps with those of senescence in endothelial and dental pulp cells (83, 84) and in bile duct cells of patients with biliary cirrhosis (85, 86). Increased autophagic activity is responsible for the death of senescent keratinocytes (87, 88). A study examining the direct relationship between autophagy and oncogene-induced senescence (OIS) by Young et al. showed that autophagy speeds up senescence, although once set in, the latter could not be reversed by blocking the former (89). This was also verified in post-chemotherapy senescent cancer cells (90). At a molecular level, both phenomena are controlled by overlapping signaling pathways involving ROS generation, DDR activation, p53 and p21 tumor suppressor induction (89, 90). It is possible that autophagy is induced to help cells produce energy in anticipation of the senescence-associated growth arrest (80). Some reports suggest an inverse relationship between autophagy and senescence, such that the inhibition of the former facilitates the latter, particularly in the context of oncogene and chemotherapy-induced senescence (91–93). This could be explained if senescence serves as a backup for cells failed to initiate autophagy to survive external and internal stressors (80). Nevertheless, to firmly decide whether or not autophagy is essential for senescence induction, more experiments are required, such as those i) conducted in cells with defective apoptosis, ii) involve spatial and temporal regulation of autophagy and senescence, and iii) consist of careful monitoring of the two simultaneously (80).

(I) Classically, the p53/p21 and p16/RB tumor suppressor pathways are responsible for the induction and maintenance of senescence (94). According to recent literature however, senescence is a form of stress response influenced by many effector pathways (41). Different phenotypic changes autonomous or non-autonomous to the senescent cells, are triggered by specific combinations of these effector programs. For example, the DDR and SASP trigger autonomous changes in senescent cells *via* effector signaling pathways p38 and PI3K/AKT/mTOR and chromatin level alterations such as formation of SAHF and PML bodies. The non-autonomous/paracrine changes to the senescent microenvironment are mediated through SASP by affecting immune response, fibrosis, wound healing, angiogenesis, cellular plasticity, etc. Activation of chronic DDR signaling pathway is important for SASP production and depletion of several DDR-associated proteins negatively affects expression of several key SASP components such as IL-6, IL-8, and GRO family members. Transcription factors NF-κβ and CCAAT enhancer-binding protein β (C/EBPβ) are involved in global regulation of SASP constituents (41, 94, 95).

Interestingly, the long list of detrimental effects of senescence as it pertains to aging and age-related diseases poses a fundamental question: why would such a maladaptive process evolve in human, especially when many organisms do not experience aging/senescence (96)? This paradox is a topic of intense discussion and outside the scope of this review. According to the most straightforward theory, senescence induction is selected for in early life to prevent accumulation of damaged cells and support healing following injuries. As the force of natural selection decreases with age, the efficiency of senescence cell clearance reduces and its adverse effects become evident. This is comparable to the antagonistic pleiotropy theory of aging which posits that natural selection drives evolution by selecting genes that provide early life benefit to maximize reproductive fitness, but once the reproductive period ends, the organism enters a window of weakened selection leading to hyper-inflammation, immune evasion, tumor promotion, and other age-related disorders (97, 98).

## Senescence in Malignant Transformation and Cancer Therapy

Malignant transformation is characterized by uncontrolled cellular proliferation through gain of oncogenes or loss of tumor suppressors (99). However, it does not always lead to overt cancer, as is the case with dormant benign tumors, such as melanocytic nevi exhibiting proliferative arrest (100). One of the contributing factors to this phenomenon is oncogene-induced senescence/OIS, first reported with the *Ras* oncogene-transformed human primary lung fibroblast IMR90 and mammary epithelial cells (101, 102). OIS, stimulated by activated oncoproteins or inactive tumor suppressor proteins such as BRAF, RAS, AKT, E2F1, cyclin E, PTEN, or NF1, occurs prematurely in absence of telomere shortening (103), however, depends on the extent of oncogene overexpression (104). Persistent DNA damage, tumor suppressors p53, pRB, and several microRNAs are key regulators of OIS (103). In addition to growth arrest, OIS is characterized by SA-β-gal activation, SASP production, and stimulation of autophagy (103).

It is believed that OIS is a fail-safe tumor-suppressive mechanism (105). However, it can also be tumor-promoting (106), particularly through the involvement of different SASP components (68, 103). For example, TGF-β and MCP1 propagate growth-arrested phenotype in the neighboring non-senescent cells (107), while MCP1 and CXCL1 promote immune clearance of senescent cells by attracting NK cells and tumor-suppressive M1 macrophages (108, 109). Again, VEGF, IL-6, IL-8, and CXCL1 support tumorigenesis through their positive effects on angiogenesis, invasion, and CSCs (110–113). By recruiting M2 macrophages and immature myeloid cells, MCP1 is able to create an immunosuppressive environment in the vicinity of the senescent cells, helping them to escape immune-clearance (114, 115).

As the long-term presence of senescent cells from OIS can promote tumorigenesis, their direct removal or prevention of SASP production is perceived as a tumor-protective strategy. Two major classes of therapeutic agents targeting senescent cells are available: senocidals (further categorized as senolytics and senotopics) and senomorphics. These include varieties of natural products, endogenous compounds, investigational and approved drugs [(116) and references therein]. Senocidals eliminate senescent cells by promoting apoptotic (senolytics) or non-apoptotic (senotopics) cell death, while senomorphics suppress SASP production. However, given the dynamic and complex nature of senescence and SASP, extensive testing is needed for any of the compounds to be useful in clinical settings. Additionally, because SASP stimulates tumor-suppressive immune-clearance of senescent cells, a senescence inducing/pro-senescent therapy is also being investigated in various cancer types including BC (117). Diverse types of agents such as targeted and chemotherapeutic drugs, phytochemicals, and epigenetic modulators are being examined for this specific purpose (117).

One side effect of suppressing SASP production is that it may cause senescent cells no longer recognizable by the immune system, persist over a long-period of time, eventually interfering with healthy tissue function (118). Targeting specific SASP components with neutralizing antibodies, for example, could help avoid this problem. Likewise, senescent cells, induced by pro-senescence therapies, unless rapidly cleared by the immune system, could essentially accumulate, altering the tumor and immune microenvironment through persistent SASP production. This in the long-run might result in tumor relapse and metastasis (119). Another likely side effect of pro-senescence therapy when administered *via* systemic route is due to the generation and accumulation of senescent cells in different tissues and organs, which in turn might accelerate the onset and progression of chronic aging-associated disorders such as cardiovascular, neurodegenerative, fibrotic diseases, to name a few (120, 121). Some of these detrimental effects of pro-senescent therapies can be overcome by careful selection of the therapeutic agents, choosing appropriate delivery routes, continuous monitoring of the therapy response, and using adjuvant immunotherapy preventing tissue build-up of senescent cells (118, 119, 122).

## TNBC and Chemoresistance

TNBC, lacking expression of the estrogen, progesterone receptors (ER, PR) and amplification and/or overexpression of the Human Epidermal Growth Factor Receptor 2 (HER2), is characterized by high mitotic index, advanced grade and stage, and increased immune cell infiltration. TNBC patients often experience poor prognosis, frequent distant metastases, recurrent disease, and reduced overall survival (9, 10, 13). TNBCs are further categorized into several molecular subtypes (basal-like: BL1 and BL20, immunomodulatory: IM, mesenchymal: M, mesenchymal stem-like: MSL, luminal androgen receptor: LAR), with each sensitive to specific classes of drugs (8).

Because of extensive heterogeneity and lack of HER2, ER, PR expression, chemotherapy is the most preferred choice of treatment for TNBC patients (10, 123). In neoadjuvant setting, chemotherapies used are primarily anthracyclines and taxanes, while in adjuvant setting, much diverse combinations consisting of anthracyclines, taxanes, cyclophosphamide, methotrexate, 5-Fluorouracil, gemcitabine, and vinorelbine are prescribed (10–12). Despite better initial complete pathological response (pCR) than other BC subtypes, especially in the neoadjuvant setting, emergence of resistance is a common phenomenon responsible for poor clinical outcome in TNBC (11, 12).

There are detailed reviews discussing different modes of chemoresistance in TNBC available (10, 17, 19). Briefly, these are altered expression of ATP-binding cassette (ABC) transporters and microRNAs, heightened drug metabolism, evasion of apoptosis, enrichment of cancer stem cells and related signaling pathways (especially those associated with embryo development), induction of DNA damage and inflammation, activation of lipid kinase and tyrosine kinase signaling pathways, hypoxia, tumor-suppressive immune environment, and inherent intra and inter-tumoral heterogeneity (10, 17, 19). Although, each of these are potential therapeutic target, because of their complicated interactions and collaborations, they need to be thoroughly studied before bringing into the clinic. Recent trials have confirmed an urgent need for combination treatment and biomarker-based patient selection strategies to enhance the cancer cell specificity and selectivity and lower systemic toxicity. In this regard, two types of therapeutic interventions, metronomic chemotherapy and polychemotherapy, are particularly noteworthy. The former involves frequent administration of chemotherapeutic drugs below the maximum-tolerated dose (MTD), while the latter utilizes combinations of several drugs. However, none of these has been approved yet (124–127).

In addition to individual molecules or signaling pathways, specific cellular fate that TNBC cells readily adapt to avoid chemotherapy-induced cell death, also contributes to drug resistance and eventual disease recurrence. Notable among these is therapy-induced senescence (TIS) (10). In addition to direct response to chemotherapies, TIS may also be prompted by microenvironmental stressors including hypoxia, nutrient deprivation, and oxidative damage, which in turn alter patterns of chemotherapy response (128–131). There are numerous reports of occurrence of TIS in BC cell lines, including those of TNBC origin by standard genotoxic agents including doxorubicin (132, 133), etoposides (134), irinotecan (132), methotrexate (132), paclitaxel (132, 135), cisplatin (136, 137), and even with metronomic schedule (138). In clinical setting, Poele et al. was one of the first to report presence of senescent cells in archival samples of breast tumors from patients receiving neoadjuvant chemotherapy (cyclophosphamide, Adriamycin, and 5-fluorouracil). Compared to the 10% samples from patients who received no treatment prior to surgery, 41% chemotherapy-treated tumors showed SA-β-gal-positivity. The authors also found an association between SA-β-gal staining with low p53 and high p16 staining. Normal tissue sections or normal cells surrounding the tumor sections were completely negative for SA-β-gal and did not have altered expression of the above-mentioned two tumor suppressor proteins. They concluded that senescence induction is a natural response to chemotherapy treatment in BC and it may play important role in determining treatment outcome (139). Another study that discussed the importance of TIS in disease prognosis and therapy response was by Laine et al., who demonstrated that overexpression of CIP2A (cancerous inhibitor of PP2A), a negative regulator of senescence leads to adverse patient outcome and resistance to senescence-inducing chemotherapy (140). Using genetically engineered model of mouse mammary tumor (*MMTV-Wnt1*) Jackson et al. established a detrimental association between senescence and chemotherapy response, specifically in the wild-type (WT) p53 background (a key regulator of senescence), possibly through SASP (141). Their data corroborated previous reports indicating a negative association between functional p53 and response to high dose chemotherapy in patients with advanced BC (142, 143). It also provided an explanation to the fact that majority of basal-like BC (included into the TNBC subtype) (144) with mutated p53 exhibits complete response to chemotherapy, while the luminal subtype retaining WT p53 is somewhat chemoresistant (18, 145). Instead of examining the effect of adjuvant chemotherapy on tumor cells, Sanoff et al. focused on non-malignant cells in BC patients. They discovered a 75% increase in p16 mRNA levels in peripheral T cells, which was accompanied by a stable increase in the levels of two SASP components VEGF and MCP-1 in patients’ plasma. While the majority of patients displayed signs of accelerated molecular aging that sustained until several years after therapy, the response was highly variable. They also discovered that the post-chemotherapy molecular aging is equivalent to 10–15 years of chronological aging. The authors concluded that such detrimental side effects of chemotherapy is responsible for the long-term systemic toxicity in cancer patients whose magnitude depends on the molecular rather than the chronological age of the individual (146, 147). This was corroborated by another study that implicated SASP components (IL-1α, IL-6, IL-8, CCL2, and CXCL12) in the short and long-term comorbidities of chemotherapies, for example fatigue, cardiac dysfunction, reduced bone volume and density, loss of physical functions and appetite (148).

A handful of studies reported a two-step strategy therapeutically exploiting TIS in TNBC pre-clinical models. The first step involved induction of TIS with chemotherapy or other treatment modalities, while the second step consisted of follow-up treatment with senolytics. For example, Galiana et al. explored the effect of palbociclib-induced TIS, followed by senolysis with nano-encapsulated navitoclax in immunocompetent mouse models of advanced TNBC and discovered tumor growth inhibition and reduced metastasis (29). In another study, TNBC cell lines were successfully inhibited by sequential treatments with senescence-inducing BET domain inhibitor and senolytic navitoclax (149). A discovery-stage biopharmaceutical company Senolytic Therapeutics (STX) is currently developing a diagnostic test SenolT for detecting and monitoring post-therapy (radiation/chemotherapy) senescent cells in liquid biopsy samples from TNBC patients (https://cordis.europa.eu/project/id/826909). The test is meant to find the association between TIS and TNBC recurrence.

## Senescence-Induced Stemness and Its Therapeutic Implication in Cancers Including TNBC

Survival of a rare population of tumor cells possessing CSC-like characteristics following chemotherapy is a key contributor of resistance (150–153). In a seminal paper published by Bhola et al., gene expression analysis of matched pair of 17 pre- and post-chemotherapy primary BC biopsies (including TNBC specimens) revealed an enrichment of signatures of CSC and TGF-β, the cytokine famous for its association with breast stem cells and CSCs in treated samples. They went on to demonstrate a causal association between post-chemotherapy CSC enrichment with TGF-β signaling, which upon pharmacological intervention prevented *in vivo* tumor relapse in pre-clinical modes of TNBC (154). While this study did not demonstrate any connection between TIS and CSC enrichment, the senescence-promoting autocrine/paracrine role of TGF-β signaling in aging/aging-related pathologies, particularly in the context of stem cells is already known (155).

Acquisition of stem-like properties following TIS induction is implicated in drug-resistance (4, 5, 20). For example, Milanovic et al. observed by using GMM models of B-cell lymphoma a substantial upregulation of stem cell signature, activated Wnt signaling pathway and stemness-associated marker expression in chemotherapy-induced senescent population (31). Induction of senescence-associated stemness (SAS) was extended beyond TIS as they detected it in the models of replicative as well as stress-induced senescence. Finally, in blood cancer cell lines and patient samples such SAS induction was found to be correlated with relapse of aggressive tumors (31). In an attempt to find out what triggers SAS, these authors and others discovered the involvement of cell-intrinsic mechanisms such as activation of Wnt signaling (31, 156) and epigenetic mechanism (157). However, SASP, particularly its pro-inflammatory cytokine constituents known to cause cellular reprogramming, plasticity, and tissue regeneration (113, 158, 159), also contributes to SAS induction (160), not only in cell-autonomous fashion, but non-autonomously by interacting with the non-senescent cells in the microenvironment (4, 5, 160). Specifically for TNBC, very few reports establishing a positive link between SAS and chemoresistance are available. The most noteworthy of these is the work reported by Achuthan et al., in which by using TNBC pre-clinical model the authors demonstrated a causal relationship between TIS and generation of chemoresistant stem-like population (20). Another study with TNBC biopsy samples added an interesting factor, polyploidy to the SAS and chemoresistance connection. The authors found that all tumors that failed to respond to neoadjuvant chemotherapy possessed a significant proportion of senescent cells (161).

In the previous section, we have already discussed the two-step strategy (senogenics, followed by senolytics treatment) for prevention/elimination of chemoresistance and relapse of aggressive, metastatic tumors. Some scientists exploring this approach also presented convincing evidences for the importance of SAS inhibition in this context (31–36). Nevertheless, conveying this observation to the clinic requires careful optimization of the dose and treatment regimen.

## Effect of TIS on the Efficacies of Immune- and DDR-Directed Therapies in TNBC

In recent years, immunotherapy has emerged as one of the most sought-out treatment strategies, capable of producing durable anti-tumor responses. Success of immunotherapy in general, depends on the inherent immunogenicity of the tumor. Although, traditionally perceived as an immunologically “cold” type, BC, especially the TNBC subtype, is now being considered curable by immunotherapies (21). In this regard, the immune checkpoint inhibitors (ICI) targeting the negative regulators of T cell activation (cytotoxic T lymphocyte-associated protein-4/CTLA-4, programmed cell death protein-1/PD-1, and programmed death-ligand 1/PD-L1), have gained the most attention. In 2019, both US FDA and European Medicines Agency (EMA) granted accelerated approval for use of the anti-PD-L1 antibody atezolizumab with nab-paclitaxel as the first-line treatment for PD-L-1+, unresectable, locally advanced or metastatic TNBC (21). A substantial number of trials exploring efficacies of PD-1 or PD-L1 antibodies against BC either as monotherapies or in combination with the radiation, chemo, targeted, or other forms of immunotherapies are in progress. Although promising, only a small percentage of TNBC patients experience a durable objective response to ICI regimen. Also, a strong tumor-associated PD-L1 signal does not always faithfully predict the overall survival, prognosis, and response to anti-PD-L-1 therapy in TNBC (21). Recent research has also indicated a detrimental role of aging and inflammation-associated effector T cell senescence in immunotherapy efficacy. A potential role of senescent T cell-derived SASP in modulation of the tumor microenvironment (TME), although not entirely clear, is suspected [(162) and the references therein].

In early stage, locally advanced or metastatic TNBC patients, chemotherapies when combined with the ICI blockage, produced encouraging anti-tumor response. This was different from the immunosuppressive effects of some chemotherapies (163). The specific effect of pre-ICI chemotherapy on metastatic TNBC was explored in the TONIC trial that included a two-week pre-conditioning with cyclophosphamide, cisplatin, and doxorubicin prior to anti-PD-1 therapy (164). The short duration of chemotherapy was assumed to be sufficient for enhancing the anti-tumor efficacy of PD-1 blockade by promoting immunogenic death of tumor cells and implementing pro-immunogenic changes in the tumor microenvironment (TME), but inadequate to negatively affect immune cells, especially the T cells. The overall objective response rate (ORR) was 20% more than the previous trials, with the highest ORR (35%) achieved with the doxorubicin induction arm. The TONIC trial clearly highlighted the favorable effect of a chemotherapy induction step prior to ICI therapy in TNBC (164).

An important question is how does TIS fit into the aforementioned benefit of chemotherapy precondition step to the PD-1/PD-L1-based immunotherapies in TNBC? Chemotherapy triggers TIS and subsequent SASP production. The immune modulatory components of SASP promote immune cell infiltration to the tumor, which upon further activation of the immune system clears both senescent and non-senescent cancer cells (26, 120, 165). This is also supported by the fact that pro-senescent therapies, although capable of prompting tumor growth arrest, are ineffective in causing tumor regression or elimination on their own and require a two-step strategy along with a functional immune system (118, 166). Mechanistic details of the sensitizing effects of SASP on ICI-directed therapies although known for cancers of the ovary (24), pancreas (25), and melanoma (26), are yet to be identified for TNBC.

Poly (ADP-ribose) polymerase (PARP) is a DNA damage repair protein which when inhibited in cancers having defective homologous recombination (HR), such as those caused by the Breast Cancer gene *BRCA1/2* deficiency, results in synthetic lethality. This is because PARP inhibitor (PARPi) treatment results in accumulation of unrepaired DNA single-strand breaks (SSBs), which during replication and in the absence of functional BRCA1/2 are converted to lethal double-strand breaks (DSBs) (167). Deficiencies in additional HR repair proteins including MRN complex, PALB2, RAD51, RAD54, DSS1, RPA1, NBS1, ATR, ATM, CHK1, CHK2, FANCD2, FANCA, and FANCC are also synthetically lethal with PAPRi (168). Currently, PARPi are recommended for the treatment of TNBC (olaparib and talazoparib) and epithelial ovarian cancers (olaparib, niraparib, and rucaparib) harboring *BRCA1/2* mutations (22), but their long-term efficacies are variable and independent of the HR status (23, 169, 170). Although, restoration of HR and replication fork stalling are the most common mechanisms of PARPi resistance, HR-independent escape strategies are not rare (171). Recent work by Fleury et al. demonstrated that the DDR elicited by PARPi renders a TIS-like state along with production of inflammatory cytokines in both breast and ovarian cancer cells leading to tumor relapse. This was overcome by treatment with senolytic drugs, such as those that inhibit anti-apoptotic BCL-2 and BCL-XL proteins. This work confirmed TIS as a critical contributor of PARPi response and supported the importance of a two-step treatment approach with PARPi and senotherapeutics in breast and ovarian cancer cells (23).

So far, we have presented necessary evidences to convince the readers that TIS plays important roles in the chemo, immune, and DDR-directed therapeutic responses in TNBC. Nevertheless, no clear guideline exists for exploiting TIS for the benefits of patients, which could be attributed to the following factors. 1) TIS is highly dependent on the nature and extent of stress, so no two therapeutic agents will impose exactly the same type of senescence response in tumor cells. 2) There is no single property of senescent cells that can be consistently used for easy detection of TIS in clinical specimens. 3) SASP production is a highly dynamic and context-dependent phenomena. 4) Cell autonomous and non-autonomous effects of SASP on the tumor cells and their microenvironment (TME) depend on the composition of SASP at any given time. 5) Tumor heterogeneity, history of inflammation, aging among others influence the overall response of the tumor and TME to TIS. 6) Senescence in non-tumor cells triggered by systemic therapies could potentially contribute not only to the drug toxicity, but also to the reduction in therapeutic benefit. We believe that some of these complexities can be overcome by generating a broad-spectrum multi-omics-based predictive TIS-signature from TNBC cells (irrespective of the type and dose of the therapeutic agent or its exposure time) and utilizing it for making therapeutic decisions. Because, SASP is responsible for most of the detrimental effects of TIS, we speculate that senomorphics (agents that interfere with SASP), rather than senolytics in combination with appropriate immunotherapeutic drugs will be superior in generating beneficial therapeutic response in TNBC.

## Tackling TIS in TNBC From the Evolution Standpoint

Although, immunotherapy and DDR-directed therapies are gaining acceptance for TNBC treatment, due to the low number of patients benefitting from both therapeutic strategies along with the scarcity of predictive biomarkers for patient selection, combination chemotherapies continue to be the standard care for TNBC patients. Compared to other BC subtypes, newly diagnosed TNBCs are more sensitive to conventional chemotherapies. However, those patients who fail to achieve complete pathologic response/pCR are at high risk of relapse and progressively poorer responses toward second-, third-, and fourth-line treatment (7, 11, 12, 17). According to the principles of evolutionary biology, this is caused by the “competitive release” of already present, yet rare resistant clones (38). In this respect, the overall poor prognosis and survival of TNBC patients can be attributed to evolution. Alternative therapeutic strategies employing the principles of dynamic tumor evolution (known as the adaptive therapy), could certainly be crucial for suppression of drug-resistant tumor cell populations and long-term TNBC control.

Owing to their inherent heterogeneity and abilities to interact with the microenvironment in a spatio-temporal and non-linear fashion, cancers can be viewed as an open complex adaptive systems, to which perturbations (such as anti-cancer drug treatment) are expected to result in unanticipated consequences (38). However, even such unpredictable systems can effectively be controlled if appropriate therapeutic strategies are designed on the basis of their dynamic nature (38). One such strategy should focus on exploiting the phenotypic cost of resistance. A popular example is the fitness differences of the multidrug-resistant ABC transporter-expressing tumor cell population in presence and absence of drug. The strategy that exploited the lower fitness of resistant cells without the drug involved alternative treatment cycles of chemotherapy and fake drug (“ersatzdroges”), forcing the resistant cells to spend significant amount of energy in pumping the drug out rather than growing and invading (172). A similar approach can be proposed for inhibiting TNBC cells that have emerged as chemoresistant through TIS. Senescence, both in oncogene and chemotherapy-induced settings, is associated with activation of the unfolded protein response (UPR) pathway (173, 174). This could somewhat be attributed to the excessive ER stress due to increased demand of synthesis, maturation, and secretion of the SASP-associated proteins (174). Others associate this with increased oxidative stress or activation of autophagy in senescent cells (174). Nevertheless, the heightened dependency on the UPR pathway could in theory render the senescent TNBC cells vulnerable to pharmacological dysregulation of ER stress (173, 174). Reliance of the senescent cells on certain metabolic pathways (175), could similarly be pharmacologically pursued as an adaptive therapeutic strategy.

Secondly, to prevent chemoresistance in TNBC through TIS, the famous “first strike-second strike” strategy put forward by Gatenby et al. (176) can also be adapted. The premise of this approach is that strategic application of drugs or drug combinations that are otherwise not curative in appropriate sequences would mimic dynamics of background extinction of many large, diverse, and geographically scattered species (comparable to heterogeneous and disseminated cancer cells). The first strike is meant to reduce the population size and diversity of the tumor, with the following strikes causing eco-evolutionary distresses pushing the vulnerable small populations of surviving cells to extinction threshold (176). In case of TNBC, the first strike could be constituted of low-dose chemotherapy, immediately followed by immune predation of senescent cells, then followed by cancer stem cell-targeting therapy. While, none of these are capable of destroying the tumor on their own, when applied in right order, would force the small, comparatively homogeneous tumor cell populations to be exterminated. With a similar strategy proven to cure pediatric ALL, we are hopeful that it would be beneficial for long-term TNBC control.

Our final recommendation is to disrupt the dynamics of “public good games” (PGGs) in TNBC played by the senescent and non-senescent tumor and microenvironmental cells. Public goods, in general are the secretory products (growth factors, angiogenic factors, metabolic intermediates, etc.) of certain cell populations that are beneficial for the tumor as whole. In a heterogeneous tumor ecosystem, public goods producers exist in dynamic equilibrium with the non-producers (cheaters and free-riders) (177, 178). While, modeling the PGGs is not an easy task (179), the interdependency between the producers and free-riders is an exploitable feature for tumor control. In their seminal work, Archetti et al., by using experimental model of neuroendocrine pancreatic cancer, studied the dynamics of cooperation and defection between the insulin-like growth factor (IGF)-II producers and free-riders (180). In a mixed population, the producers exist in a stable equilibrium with the free-riders, which can otherwise be altered by modulating the amount of growth factor. The authors proposed that modification of the dynamics of growth factor production could be a way of stable tumor control. Such observation is highly relevant for TIS-adapted TNBC cells, as SASP production by the senescent cells is crucial for the establishment and maintenance of stem-like, immune-suppressive, drug-resistant phenotypes (68). Senomorphics, by restricting the SASP production, in principle would be useful for reversion of chemoresistance. Similar approach has been recognized as an effective therapeutic strategy against aging and age-related disorders and is under intense investigation (181).

## Conclusion

**Figure 2:**
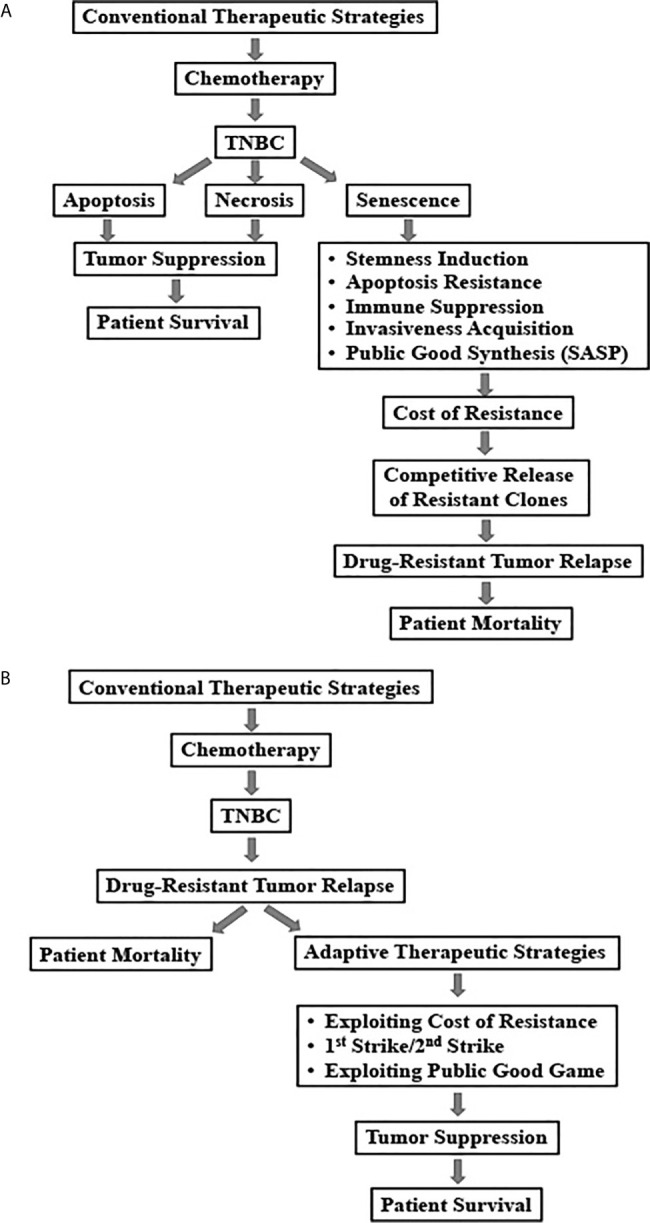
Schematic representation of conventional and adaptive treatment strategies and their outcomes in triple negative breast cancers. **(A)** Relapse of drug-resistant tumors due to conventional chemotherapy-induced senescence in TNBC patients. **(B)** Adaptive therapeutic strategies to combat chemotherapy-induced senescence in TNBC.

TNBC needs better therapeutic intervention. Even with the recent availability of the immune- and DDR-directed therapies, chemotherapy remains in the frontline of treatment choices for TNBC patients. One of the main reasons of the poor clinical outcome in TNBC patients is emergence of chemotherapy resistance. Herein, we have discussed a cellular fate, called senescence and its involvement in oncogenesis and chemoresistance, particularly in the context of induction of stemness. Finally, we have reasoned how evolution can be the major driving force of emergence of resistance and accordingly proposed three adaptive strategies to confront TIS-mediated chemoresistance in TNBC (**Figure 2**). Although, the theoretical support on TIS as an evolutionary fitter strategy is yet to be established, based on its recognition as a critical modulator of treatment outcome in cancer, we predict that soon it will receive its due attention from evolutionary biologists.

## Author Contributions

AC conceptualized the idea. AC, SC, and GC wrote the manuscript. AC, SC, and RB complied the information. All the authors contributed to the article and approved the submitted version.

## Conflict of Interest

The authors declare that the research was conducted in the absence of any commercial or financial relationships that could be construed as a potential conflict of interest.

## Abbreviations

BC, breast cancer; TNBC, triple negative breast cancer; TIS, therapy-induced senescence; SASP, senescence-associated secretory proteome; SA-βgal, senescence-associated β−galactosidase; ROS, reactive oxygen species; OIS, oncogene-induced senescence; ER, estrogen receptor; PR, progesterone receptor; HER2, human epidermal growth factor receptor; ABC, ATP-binding cassette; CSC, cancer stem cell; SAS, senescence-associated stemness; ICI, immune checkpoint inhibitor; DDR, DNA damage-response; PGG, public good game; TME, tumor microenvironment; IL-1α, interleukin-1alpha; IL-1β, interleukin-1beta; IL-6, interleukin-6; IL-8, interleukin-8; CCL2, chemokine ligand 2; CXCL1, chemokine ligand 1; CXCL12, C-X-C motif chemokine ligand 12; TGF-β, transforming growth factor beta; MCP-1, monocyte chemoattractant protein-1; GM-CSF, granulocyte-macrophage colony-stimulating factor; VEGF, vascular endothelial growth factor; PD-1, programmed cell death protein-1; PD-L1, programmed death-ligand-1; PARP, poly (ADP-ribose) polymerase; FANCA, Fanconi anemia complementation group A; FANCC, Fanconi anemia complementation group C; FANCD2, Fanconi anemia complementation group D2; ATR, ataxia telangiectasia and Rad3-related protein; ATM, ataxia telangiectasia mutated; *BRCA1/2*, breast cancer 1/2; CHK1, Checkpoint kinase 1; CHK2, Checkpoint kinase 2; DSS1, deleted in split hand/split foot 1; MRN complex, Mre11, Rad50 and Nbs1 complex; NBS1, Nijmegen breakage syndrome 1; PALB2, partner and localizer of BRCA; RAD51, RAD51 recombinase; RAD54, DNA recombination and repair protein RAD54-like; RPA1, replication protein A1; ORR, overall objective response rate; pCR, pathological complete response.
